# Intramacrophage Survival for Extracellular Bacterial Pathogens: MgtC As a Key Adaptive Factor

**DOI:** 10.3389/fcimb.2016.00052

**Published:** 2016-05-17

**Authors:** Claudine Belon, Anne-Béatrice Blanc-Potard

**Affiliations:** Laboratoire de Dynamique des Interactions Membranaires Normales et Pathologiques, Université de Montpellier (DIMNP Centre National de la Recherche Scientifique-UMR5235)Montpellier, France

**Keywords:** adaptation, extracellular bacteria, intracellular bacteria, macrophages, MgtC

Classically, pathogenic bacteria are classified as intracellular or extracellular pathogens. Intracellular bacterial pathogens, as *Mycobacterium tuberculosis, Salmonella enterica, Brucella suis*, or *Listeria monocytogenes*, can replicate within host cells. After entering the target host cell (professional or non-professional phagocyte), the intracellular pathogen follows vacuolar or cytosolic pathways to replicate. In contrast, extracellular pathogens, as *Staphylococcus aureus, Pseudomonas aeruginosa* or streptococci, avoid phagocytosis, thus promoting extracellular multiplication. However, the situation appears more complex with a dual lifestyle of intracellular/extracellular bacterial pathogens (Silva, 2012). Indeed, diverse bacterial intracellular pathogens have the ability to produce extracellular infections as a second phase after the initial intracellular stage. Conversely, several extracellular pathogens can enter host cells *in vivo*, resulting in a phase of intracellular residence, which can be of importance prior the typical extracellular infection.

During infection, macrophage lineage cells eliminate infiltrating pathogens through a battery of antimicrobial responses that include oxidative and acid stresses, toxic metal cations, and antimicrobial peptides. Bacterial pathogens have developed strategies to escape the innate immune system and counteract the microbicidal action of macrophages (reviewed by Sarantis and Grinstein, 2012). Whereas some pathogens inhibit phagocytosis (extracellular pathogens), others use virulence factors to subvert the macrophage antimicrobial role and manipulate the host-cell to establish a replication niche. Hence, intracellular pathogens overcome macrophage defenses and use these immune cells as residence and dissemination strategies. Even if it accounts for a transitory initial event, so-called extracellular pathogens can also encounter an intramacrophage stage and have therefore to escape the killing by these immune cells.

Intracellular bacteria use a broad range of molecular pathogenicity determinants to manipulate host cell processes and adapt to the intracellular environment. Recent data, described below, reveal that so-called extracellular pathogens have also acquired similar pathogenicity determinants to improve intramacrophage survival.

## Zebrafish embryos as a pertinent animal model to decipher the contribution of an intramacrophage stage during infection

The zebrafish embryo is a powerful model to examine host-pathogen interactions because it allows the study of the infection in the context of a whole living organism. Zebrafish model has contributed substantially to our understanding of the mechanisms by which different pathogens interact with macrophages and evade host innate immunity (Davis et al., 2002; reviewed by Torraca et al., 2014). The development of transgenic zebrafish lines with fluorescently labeled macrophages enable non-invasive imaging at the optically transparent early life stages to visualize the activity of phagocytic cells in real time. Furthermore, several technical tools allow specific depletion of macrophages in embryos, and offer the opportunity to analyze their contribution to the disease progression.

These powerful tools have highlighted the paradoxical roles of phagocytic cells in both limiting infection and promoting the dissemination of intracellular pathogens (Clay et al., 2007). Importantly, the role of phagocytic cells has been reconsidered for pathogens classically viewed as extracellular pathogens, as *S. aureus* and *P. aeruginosa*.

*S. aureus* has long been considered an extracellular pathogen, but there is accumulating evidence that it can also survive and replicate in phagocytes. The zebrafish embryo model has contributed significantly to the understanding of the nature and relevance of the intracellular phase in the life cycle of this pathogen (Prajsnar et al., 2012). Live imaging showed that, upon intravenous infection, *S. aureus* is completely phagocytosed by macrophages and neutrophils. Although some embryos clear the infection in a phagocyte-dependent manner, other embryos develop overwhelming infection, indicating that the bacteria can subvert the phagocyte-killing mechanisms (Prajsnar et al., 2012).

*P. aeruginosa* is an environmental bacterium and an opportunistic human pathogen that is a major cause of mortality in cystic fibrosis (CF) patients. *P. aeruginosa* is considered as an extracellular bacterium that impairs host phagocytic functions. However, infection by *P. aeruginosa* is more severe in zebrafish embryos upon phagocyte depletion (Brannon et al., 2009), which suggests a role of phagocytes in *P. aeruginosa* clearance. Indeed, neutrophils and macrophages rapidly phagocytosed and killed *P. aeruginosa* in infected embryos, supporting that phagocytic cells played a role in protection against infection. The analysis of different mutants in the zebrafish model identified the type III secretion system (T3SS) and MgtC (see below) as bacterial factors involved during infection in a macrophage-dependent manner (Brannon et al., 2009; Belon et al., 2015).

## Common adaptive strategies between intracellular and extracellular pathogens: the MgtC case

Many bacterial pathogens use secretion systems to inject virulence proteins (called effectors) directly into host eukaryotic cells to promote invasion and/or intracellular trafficking. These systems include the needle-like nanomachine type III secretion system (T3SS) (reviewed by Galán et al., 2014), the versatile type IV secretion system (T4SS) that can translocate DNA or proteins into host cells (reviewed by Voth et al., 2012) and the contractile nanomachine type VI secretion system (T6SS) deployed by many bacterial species to target either host cells or rival bacteria (reviewed by Kapitein and Mogk, 2013). Following delivery into the host cytoplasm, effectors manipulate host cell biology, such as cell signaling, secretory trafficking, cytoskeletal dynamics, and the inflammatory response. The structural components of each type of secretion system exhibit a certain degree of conservation across diverse bacteria but bacterial effectors proteins appear to be more pathogen-specific.

**Figure 1:**
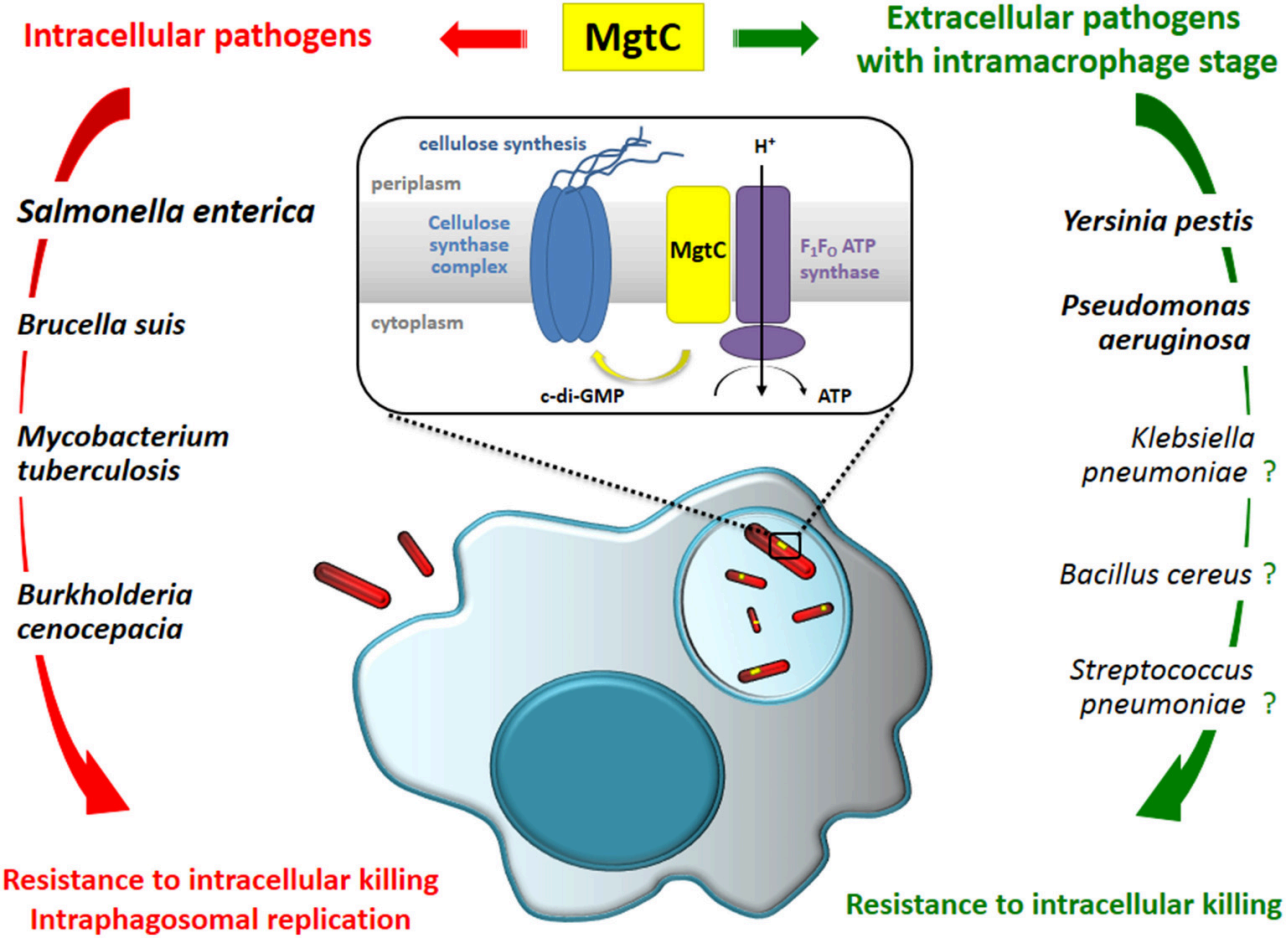
**MgtC promotes an intramacrophage phase in intracellular and so-called extracellular pathogens and may be considered as a clue to reveal bacterial pathogens adapted to an intramacrophage stage**. The contribution of MgtC for intramacrophage survival/replication has been demonstrated experimentally for several pathogens indicated in bold. We propose that it may also play an intramacrophage role in several other bacterial pathogens as *K. pneumonia, B. cereus*, and *S. pneumoniae*. Expression of *mgtC* gene has been shown to be induced inside macrophages in *S. enterica* and *P. aeruginosa*. Based on the analysis of the *Salmonella* protein, MgtC's role in macrophage has been linked to the modulation of F_1_F_o_ ATP synthase activity and to the repression of cellulose production. It is however unknown how this function is conserved for other MgtC proteins that play a role in macrophages.

In addition, several non-secreted bacterial factors, such as enzymes involved in resistance to oxidative stress, promote adaptation to the intramacrophage environment. In this regard, the MgtC virulence factor appears as an appealing factor associated with intramacrophage survival in several intracellular pathogens including *S. enterica, M. tuberculosis*, and *B. suis* (Alix and Blanc-Potard, 2007) (Figure 1). In *Salmonella*, MgtC promotes pathogenicity by inhibiting the bacterial F_1_F_o_ ATP synthase, thus hindering proton translocation and ATP synthesis (Lee et al., 2013). The inhibition of F_1_F_o_ ATP synthase activity may help the bacteria to cope with metabolic imbalances linked to the acidification of the phagosomal vacuole. Moreover, MgtC promotes *Salmonella* intramacrophage replication by repressing cellulose production during infection, through the modulation of c-di-GMP level and expression of cellulose synthase genes (Pontes et al., 2015). MgtC also promotes growth in magnesium depleted environment, but this function is distinctive from MgtC role in host environment (Rang et al., 2007). *Salmonella mgtC* is regulated both at the transcriptional and post-transcriptional levels, with a positive regulation by Mg^2+^ deprivation and increase in cytosolic ATP, and a negative regulation by the MgtR peptide (reviewed by Lee and Lee, 2015). In agreement with the intramacrophage role of *Salmonella* MgtC, expression of the *mgtC* gene is highly induced when the bacteria reside inside macrophages (Eriksson et al., 2003). The intracellular signal that induces *Salmonella mgtC* transcription is still a matter of debate, but cationic peptides and acidic pH have been proposed as potential signals (Prost and Miller, 2008).

Genes encoding MgtC-like proteins are found in a limited number of eubacterial genomes and phylogenetic analysis suggested that *mgtC* has been acquired by horizontal gene transfer repeatedly throughout bacterial evolution (Blanc-Potard and Lafay, 2003). Alignment of MgtC-like proteins as well as hydrophobicity pattern clearly define two domains: an hydrophobic N-terminal part, highly conserved in all MgtC-like proteins, and a soluble C-terminal part that is much more variable, but specifically conserved in the subgroup of MgtC proteins (referred as “true MgtC” proteins) that includes MgtC proteins from the intracellular pathogens *S. enterica, M. tuberculosis*, and *B. suis*. This phylogenetic subgroup also contains proteins from bacteria that have not been always classified as intracellular, like *Burkholderia cenocepacia*, or that are considered as mainly extracellular, as *Yersinia pestis*. *B. cenocepacia* is commonly believed to exist extracellularly in biofilms in the lungs of infected patients but many studies have shown that *B. cenocepacia* can survive and replicate inside macrophages by interfering with phagolysosomal degradation pathway (Valvano, 2015), and this intramacrophage stage has been confirmed *in vivo* using the zebrafish model (Vergunst et al., 2010). *Y. pestis* replicates mainly extracellularly and produces antiphagocytic factors but is able to survive in macrophages, which may contribute to initial infection phase. Importantly, MgtC has been shown to promote intramacrophage survival both in *B. cenocepacia* (Maloney and Valvano, 2006) and *Y. pestis* (Grabenstein et al., 2006), confirming an intracellular role even in a so-called extracellular pathogen (Figure 1).

More surprisingly, an intramacrophage role has been uncovered for *P. aeruginosa* MgtC using the zebrafish model (Belon et al., 2015). A *P. aeruginosa mgtC* mutant is attenuated for acute infection in zebrafish embryos and MgtC most likely acts by protecting *P. aeruginosa* against phagocytes since macrophage depletion suppressed the difference between *mgtC* mutant and wild-type strain. This hypothesis was supported by *ex vivo* experiments showing that the *mgtC* mutant is more sensitive to bacterial killing than the wild-type strain in cultured macrophages. In agreement with this intramacrophage role, expression of *Pseudomonas mgtC* gene is highly induced when the bacteria reside inside macrophages (Belon et al., 2015). Hence, similarly to intracellular pathogens, *P. aeruginosa* has acquired the MgtC macrophage subversion factor to resist killing by macrophages (Figure 1). It is currently unknown whether MgtC has the same biochemical function in classical intracellular pathogens like *Salmonella* and extracellular pathogens, like *Y. pestis* and *P. aeruginosa*.

## MgtC as a clue to reveal bacterial pathogens adapted to an intramacrophage stage?

Based on the current knowledge on MgtC role in various bacteria, we wish to propose that MgtC provides an unexpected tool to uncover or support the relevance of an intramacrophage stage during infection for bacterial pathogens, including so-called extracellular pathogens. For bacteria encoding MgtC-like proteins that belong to the *Salmonella* MgtC cluster (“true MgtC”) and encountering macrophages during their life-cycle, we predict that MgtC will promote survival in this hostile environment. To illustrate our viewpoint, we provide below few examples (non-exhaustives) of MgtC proteins that may contribute to intramacrophage adaptation (Figure 1).

The *Klebsiella pneumoniae* genome is one of the rare enterobacterial genome hybridizing with *Salmonella mgtC* sequences (Blanc-Potard and Groisman, 1997) and the predicted *K. pneumoniae* MgtC protein is very closely related to the *Salmonella* MgtC protein. *K. pneumoniae* is another example of a pathogen that has been largely considered as an extracellular pathogen. Interestingly, this pathogen was recently shown to survive within macrophages in a specific niche that avoids the classical endocytic pathway and the fusion with lysosomes (Cano et al., 2015). One can thus hypothesize that MgtC may contribute to the ability of *K. pneumoniae* to resist killing by macrophages.

One may also infer that the presence of *mgtC* gene in the bacterial genome of *Bacillus cereus* and in the genomes of some *Streptococcus pneumoniae* strains reveals that these strains encounter an intramacrophage stage, where MgtC may play a role. *B. cereus* is an emerging human pathogen, able to escape from macrophages after engulfment (Tran and Ramarao, 2013). *S. pneumoniae* is a pathogen that is engulfed and killed by human alveolar macrophages (Gordon et al., 2000), and strains that encode MgtC may resist better to macrophage killing than others.

Yet, we do not imply that every bacteria encoding “true MgtC” will encounter macrophages, and the presence of MgtC-like proteins in non-pathogenic bacteria may be rather related to the adaptive function of MgtC to magnesium deprivation. Conversely, some extracellular pathogens, as *S. aureus*, can reside in macrophages and lack MgtC.

## Conclusion

Host cell subversion by intracellular bacteria requires amazing mechanisms of adaptation. The increasing number of examples for host cell subversion by so-called extracellular pathogens emphasize the relevance of a transient intracellular stage during infection. The horizontally-acquired MgtC virulence factor provides a singular example of a determinant that contributes to intramacrophage adaptation both in intracellular pathogens and extracellular pathogens. By reconsidering MgtC distribution in relation with published data on bacterial lifestyles, we propose MgtC as a clue to predict bacterial adaptation to an intramacrophage stage. The identification of bacterial factors common to several intracellular and extracellular pathogens, as MgtC, provides valuable targets for new antimicrobial strategies to better control infectious diseases.

## Author contributions

AB wrote the paper and CB drew the figure.

## Funding

Vaincre La Mucoviscidose (IC0902 and RF20110600446/1/1/47) and Association Gregory Lemarchal.

### Conflict of interest statement

The authors declare that the research was conducted in the absence of any commercial or financial relationships that could be construed as a potential conflict of interest.
